# FAIRHiveFrames-1K: A Public FAIR Dataset of 1265 Annotated Hive Frame Images with Preliminary YOLOv8 and YOLOv11 Baselines

**DOI:** 10.3390/s26082518

**Published:** 2026-04-19

**Authors:** Vladimir Kulyukin, Reagan Hill, Aleksey Kulyukin

**Affiliations:** 1 School of Computing, College of Engineering, Utah State University, Logan, UT 84322, USA; 2 Department of Mathematics and Statistics, Utah State University, Logan, UT 84322, USA

**Keywords:** FAIR datasets, precision apiculture, Agriculture 4.0, image-based hive frame analysis, image-based hive comb analysis, object detection, YOLO, *Apis mellifera*

## Abstract

In precision apiculture, the portable digital camera is a cost-effective sensor for capturing hive images or videos used to quantify different colony variables. Openly accessible, well-annotated, interoperable cell-level image datasets are still the exception rather than the norm. This shortage constitutes a major barrier to AI-driven approaches aimed at automating image-based comb analysis. In this article, we present FAIRHiveFrames-1K, a publicly available dataset of 1265 annotated hive frame images (1920 × 1080 PNG) designed to facilitate research in AI-intensive image-based comb analysis automation. The dataset, derived from a 2013–2022 U.S. Department of Agriculture–Agricultural Research Service multi-sensor research reservoir, includes 124,669 annotated regions of interest for seven biologically meaningful categories consistent with comb analysis literature and standard hive inspection protocols. FAIRHiveFrames-1K is curated according to FAIR principles (Findable, Accessible, Interoperable, Reusable) and distributed under CC-BY 4.0 with standard annotation formats, fixed training and validation splits, and reproducible benchmarking artifacts. To establish preliminary baseline performance, we iteratively tuned four YOLO architectures (YOLOv8n, YOLOv8s, YOLOv11n, YOLOv11s) under a shared tuning protocol over the period of dataset growth.

## 1. Introduction

The managed honey bee (*Apis mellifera*) is fundamental to local and global agricultural systems, because the pollination services it provides are annually valued at approximately $20 billion within the United States alone [1]. Recent surveys of commercial beekeeping operations report substantial annual colony losses [2,3], consistent with longer-term evidence of widespread pollinator declines over the past decade and a half [4]. Colony decline is associated with multiple stressors, pathogens, and their interactions [5]. Understanding how colony development responds to these interacting stressors is a priority for research and commerce. In assessing colony strength, experienced beekeepers track parameters such as the number of bees on a frame, the amount of capped and uncapped brood, honey stores, and pollen reserves [6]. These field estimates are valuable but also time-consuming and expensive. The slow, episodic, and error-prone nature of the obtained results has motivated researchers to reduce colony size in controlled experiments on stressors [7,8]. However, the results obtained from small colonies may not scale to full-size colonies, because the number and diversity of interactions among colony members increase substantially with colony size [9].

In precision apiculture, the portable digital camera is a cost-effective sensor for capturing hive images or videos used to quantify various colony variables. Digital photographs of hive frames taken during routine hive inspections provide a practical approach for supplementing manual assessments [10]. Images and videos captured by on-hive cameras serve as reliable resources for longitudinal forager traffic monitoring at the hive’s entrance [11,12]. Images and videos can be stored as permanent data records, shared with colleagues, evaluated later in the laboratory or, equipment permitting, directly in the field, and integrated with other sensors such as hive scales and in-hive temperature probes [13]). Sustainable progress in precision apiculture systems that use digital cameras depends not only on model innovation but also on openly available, well-annotated datasets completely decoupled from specific models, because such decoupling is fundamental for cross-model comparison.

Semi-automated image-based comb analysis methods have been proposed to estimate capped brood cells, capped honey cells, or uncapped nectar cells, using tools including Photoshop [10], ArcView [14], and ImageJ [15]. CombCount [16], another semi-automated tool for hive frame analysis, is an open-source Python application for measuring capped brood and capped honey areas in digital frame images. The CombCount user manually delineates capped brood and capped honey regions, and the system estimates their areas.

Recent advances in machine learning and deep learning have resulted in the application and adaptation of AI-intensive methods for colony health monitoring [17]. E.g., DeepBee© is an open-source system designed to detect comb cells and classify their contents into seven categories (*Eggs*, *Larvae*, *Capped Brood*, *Pollen*, *Nectar*, *Honey*, and *Other*) [18]. A recent study showed the feasibility of AI-driven, cell-based diagnostics in distinguishing brood cells infected with European foulbrood from viral infections [19].

Successful investigations of semi-automated and automated cell-level hive frame analysis suggest that reducing the time and cost of colony-level monitoring through automation is a more scalable long-term strategy than reliance on studies focused on individual bees or experimentally reduced colonies, along with acceptance of their inherent limitations. However, progress is constrained by the relative scarcity of large, well-annotated FAIR (Findable, Accessible, Interoperable, Reusable) datasets suitable for reproducible evaluation, because scalable AI-driven methods depend on them for quality control and comparative evaluation [20]. In precision apiculture, openly accessible, well-documented, interoperable cell-level datasets are still the exception rather than the norm [21]. This shortage constitutes a major barrier to reproducible benchmarking and methodological improvement in AI-driven image-based comb analysis. A principal objective of this article is to work toward overcoming this barrier by introducing FAIRHiveFrames-1K, a FAIR, publicly available cell-level annotated dataset of hive frame images accompanied by pilot YOLOv8 and YOLOv11 baselines on this dataset (cf. the Data Availability Statement after the main text of our article).

The principal contributions of our article are:

- We introduce a large cell-level annotated image dataset to support the automation of image-based hive comb analysis.
- We document a multi-annotator consensus-based protocol and the practical labor requirements and challenges of dense cell-level hive frame image annotation.
- We report conservative baseline benchmarks obtained through an iterative model tuning process aligned with dataset growth (September 2024–December 2025).
- We analyze per-category model detection performance under standard COCO-style evaluation to expose how sparse labeling in dense hive comb images systematically affects evaluation metrics.

Investigations of fully automated image-based hive frame analysis are relatively rare in the precision apiculture literature. DeepBee© [18] is an important exception and contribution, most relevant to our work. DeepBee© is an integrated deep learning pipeline for comb cell detection and classification. Our contribution differs in method and emphasis (cf. Table 1). Rather than proposing a new integrated application, we focus on dataset engineering, reproducible benchmarking, complete decoupling of model and data, and FAIRness.

The remainder of our article is organized as follows. In Section 2, we provide the scientific metadata for FAIRHiveFrames-1K. In Section 3, we document the image capture and annotation methods and FAIR alignment. We provide the details of tuning YOLOv8 and YOLOv11 on FAIRHiveFrames-1K to generate preliminary conservative baselines. In Section 4, we provide and interpret our tuning results. In Section 5, we place our results in a broader context and discuss limitations and alternatives. In Section 6, we conclude with future research directions that we have identified in curating our dataset.

## 2. Scientific Metadata

FAIRHiveFrames-1K originates from a longitudinal digital reservoir collected between 2013 and 2022 by the Carl Hayden Bee Research Center (CHBRC) of the U.S. Department of Agriculture’s Agricultural Research Service (USDA–ARS) in Tucson, Arizona, USA, in collaboration with academic and commercial partners. This reservoir comprises multi-year, multi-site field experiments with managed honey bee colonies in Arizona, Mississippi, Arkansas, North Dakota, Idaho, California, and Australia (Table 2).

The publications in Table 2 are peer-reviewed scientific metadata for the reservoir that includes digital records of continuous hive weight measurements, in-hive temperature recordings, CO_2_ monitoring, visual hive assessments, agrochemical residue analyses, microbiome characterizations, and thousands of digital hive frame photos [39]. Below we briefly outline several major research themes in these publications.

**Continuous Sensor-Based Colony Monitoring:** A central theme is high-frequency, continuous monitoring of managed colonies with electronic hive scales and in-hive temperature probes [22,24,29]. Piecewise regression and related modeling techniques were used to quantify within-day dynamics and thermoregulation [28,29]. Detrended within-day weight variability correlated with adult bee mass, brood production, and foraging effort, while internal temperature variability served as an indicator of brood presence and colony strength. Several experiments incorporated CO_2_ monitoring and newly emerged bee dry weight measurements to refine colony-level diagnostics [34].

**Chemical Stressor Experiments:** Exposure of colonies to imidacloprid was evaluated in diverse environments over multiple years [23,25,32,36]. Additional studies investigated methoxyfenozide [31], clothianidin [34], and flonicamid [35]. These experiments combined controlled syrup or pollen patty exposure with continuous sensor monitoring and periodic hive inspections. Sublethal pesticide exposure often altered behavioral metrics derived from weight and temperature data without always producing large changes in adult bee mass or brood area. Treatment effects frequently depended on environmental context, forage availability, and year-to-year variability.

**Landscape, Forage, and Migratory Pollination Effects:** Several investigations examined how landscape composition and forage availability influence colony performance and physiology. Colonies located near U.S. Conservation Reserve Program lands exhibited improved brood production, adult bee populations, and expression of nutrition- and immunity-related biomarkers compared to colonies in intensive agricultural landscapes [30]. Supplemental winter forage prior to almond pollination improved colony survival and altered queen pheromone signaling [26,27]. Additional studies tracked commercial colonies across distinct original landscapes and major pollination events in California, demonstrating that initial landscape exposure influenced colony growth, thermoregulation, agrochemical residue profiles, and subsequent pollination performance [33].

**Colony Management Interventions:** Several investigations examined hive entrance orientation effects on colony winter activity and thermoregulation [37] and assessed effects of short-term cold storage as part of integrated Varroa management [38]. These interventions were evaluated using the same continuous sensor-based framework described above, allowing direct comparison with chemical and landscape experiments.

**Digital Hive Frame Photography:** In parallel with sensor monitoring, periodic manual hive inspections were conducted during these investigations in order to estimate adult bee mass, brood area, honey stores, and pollen reserves. As part of some inspections, high-resolution digital photographs of hive frames were captured and archived. These images were originally used for manual and semi-automated brood surface area estimation, documentation of experimental conditions, and validation of sensor-derived metrics. FAIRHiveFrames-1K is the first annotated image dataset derived from this image reservoir. We had no control over the original image acquisition. Thus, our dataset is not the outcome of a controlled data collection workflow, but rather a retroactively curated subset of an existing data source.

## 3. Materials and Methods

### 3.1. Image Acquisition

**Figure 1:**
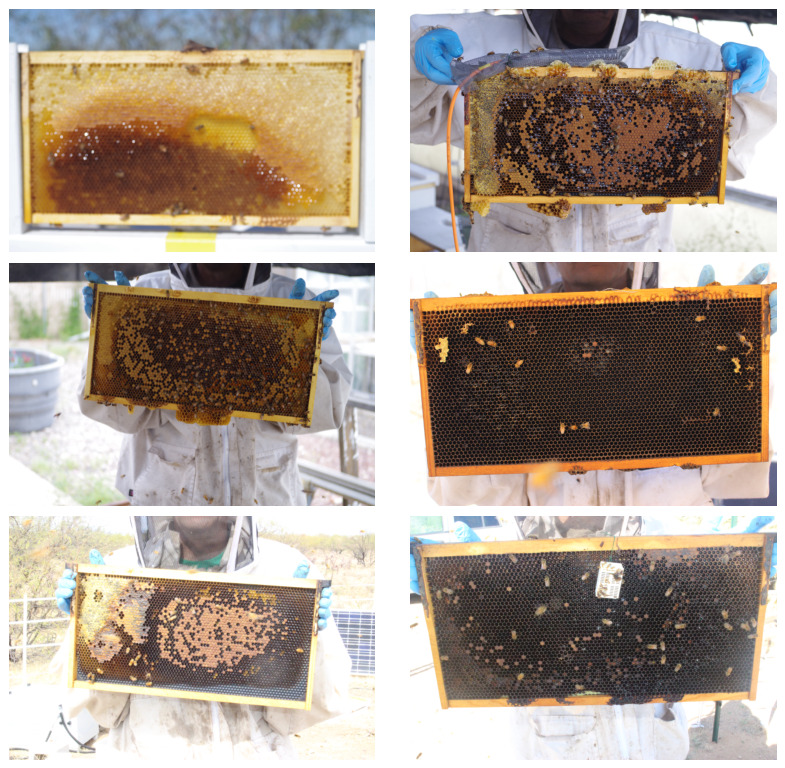
**Six images from the CHBRC Image Reservoir.** Images were acquired during hive inspections using Canon EOS Rebel SL1 (**left column**) and PENTAX K-01 (**right column**). JPEGs were saved at 5184 × 3456 (Canon) or 4928 × 3264 (Pentax).

All monitored hives consisted of two deep Langstroth supers. During inspections, each hive was opened, frames were removed, and bees were gently brushed from both sides before imaging. Both sides of each frame were photographed using a digital camera (Canon EOS Rebel SL1 or PENTAX K-01, Tokyo, Japan). Images were saved in JPEG format at one of two resolutions: 5184 × 3456 (Canon EOS Rebel SL1) or 4928 × 3264 (PENTAX K-01). Minimal field standardization was applied to camera-to-frame distance, illumination, background, or frame orientation. Figure 1 shows a six-image sample from the original reservoir.

**Figure 2:**
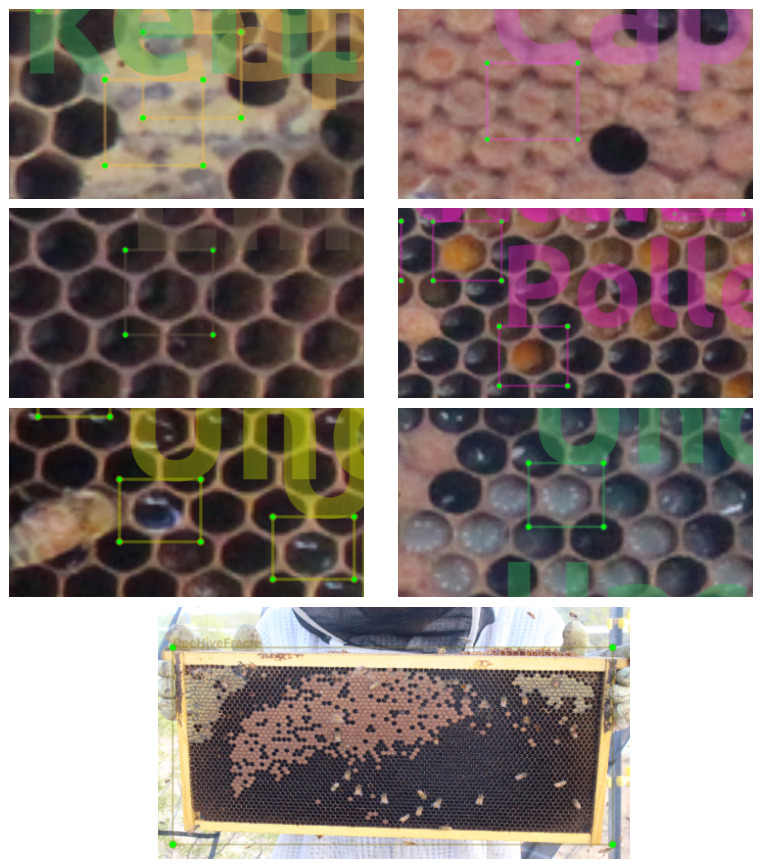
**Seven categories annotated as rectangular ROIs with** **labelImg****.** Row 1 (left)—CappedHoneyCell; Row 1 (right)—CappedWorkerBroodCell; Row 2 (left)—EmptyCombCell; Row 2 (right)—PollenCell; Row 3 (left)—UncappedNectarCell; Row 3 (right)—UncappedWorkerLarvaCell; Row 4 (center)—BeeHiveFrame.

All 1265 images selected for FAIRHiveFrames-1K were resized to 1920 × 1080 PNG format using ffmpeg on Ubuntu 18.04 LTS. The resized images were annotated in labelImg 1.8.6 using bounding boxes for seven categories: (0) *CappedHoneyCell*; (1) *CappedWorkerBroodCell*; (2) *EmptyCombCell*; (3) *PollenCell*; (4) *UncappedNectarCell*; (5) *UncappedWorkerLarvaCell*; and (6) *BeeHiveFrame*. Figure 2 provides representative examples of all seven categories. The categories correspond to visually distinguishable states of comb usage by the bee colony described in Table 3 rather than purely geometric or pixel-level features.

The seven categories in FAIRHiveFrames-1K are consistent with standard hive frame inspection practices in the apicultural literature [6] and with the prior art on image-based hive frame analysis (e.g., [10,14,15,16,18]). The selection of these categories was further informed by our extensive consultation with USDA-ARS researchers at Carl Hayden Bee Research Center of USDA–ARS in Tucson, Arizona, USA, and the Thriving Beehives Program at the Utah State University Botanical Center in Kaysville, Utah, USA (cf. Acknowledgments).

Distinguishing between visually similar categories (e.g., *CappedHoneyCell* vs. *CappedWorkerBroodCell*) relies on a combination of color, texture, reflectivity, and biological context. These distinctions made by experienced beekeepers during manual hive inspections are difficult to quantify, because they are not defined by strict pixel-level cues but by biologically meaningful visual cues interpreted within the context of hive inspection. Therefore, some degree of cell-level annotation ambiguity is inherent, particularly under variable frame illumination and orientation, occlusion by bees, and partial cell visibility.

### 3.2. Image Annotation

Image annotation was consensus-based. All box annotations produced by trained annotators (the second and third authors—RH and AK) were reviewed by an experienced beekeeper (the first author—VK) with over 15 years of practical beekeeping experience. During this review stage, category assignments and bounding box placements were adjusted as needed to ensure compliance with standard hive inspection practices. Ambiguous cases were discussed with a USDA–ARS research entomologist or a certified Master Beekeeper affiliated with the Utah State University Extension program (cf. Acknowledgments). This additional layer of expert consultation was used selectively and sparingly due to the limited availability and high cost of professional beekeeping expertise.

Final annotation decisions were made by consensus in that, if agreement between the first author and at least one external consultant on a category could not be reached, the corresponding annotation was discarded. This conservative strategy was adopted to minimize the inclusion of potentially incorrect labels at the expense of reduced annotation density. This annotation strategy differs from independent multi-annotator labeling protocols commonly used in computer vision benchmarks. Instead of measuring inter-annotator agreement, we emphasized expert-guided consensus to ensure practical relevance in the context of hive inspection. At the same time, expert-guided consensus is a practical limitation, as qualified cell-level analysis expertise is costly and relatively scarce.

Sustainable annotation throughput was limited to approximately 5 images per annotator per day, reflecting the cognitive demands of dense small-scale cell-level labeling and category discrimination. Annotated filenames encode the original acquisition date (year, month, day), location (cf. Table 4 for location abbreviations), research subject when available (cf. Table 2), reservoir image ID (e.g., IMG_3911), and annotator initials. E.g.,

2021_09_27_RR_ColdStor_IMG_3911_VK_RH.PNG

indicates that the image was acquired on 9/27/2021 in Red Rock, Arizona, USA, as part of a cold storage (ColdStor) study, and annotated by the first author (VK) and the second author (RH). All images with no annotator initials were annotated by the first author (VK).

### 3.3. Summative Dataset Characterization

**Figure 3:**
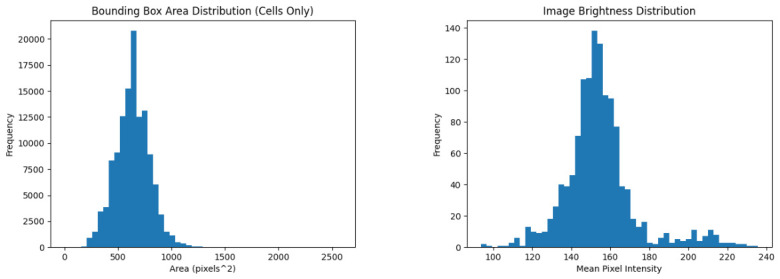
**Box area and brightness frequency histograms of FAIRHiveFrames-1K.** (**Left**): Annotation box area distribution in pixels; (**Right**): Image brightness distribution.

Table 5 gives the per-category counts of all annotated regions of interest (ROIs). The overall distribution of bounding box areas is skewed due to the coexistence of two structurally distinct objects: dense small-scale cell-level instances (categories 0–5) and the large-scale instances of the *BeeHiveFrame* category (cf. Table 6). When considered separately (cf. Figure 3 (Left)), cell-level objects (categories 0–5) exhibit a concentrated size distribution with a median area of approximately 624 pixels^2^ and low variance, corresponding to typical bounding box dimensions of roughly 25–30 pixels per side. The distribution of the cell-level pixel areas is approximately unimodal and symmetric across the dataset. In contrast, *BeeHiveFrame* occupies a substantially larger spatial scale, with bounding box areas on the order of $10^{6}$ pixels^2^, producing the tailed aggregate distribution.

Analysis of image brightness in Figure 3 (Right) shows a unimodal distribution centered around mid-range intensity values, with moderate variability across images. Most images fall within the intensity band from 115 to 179, while a smaller number of darker and brighter cases reflect larger variation under field acquisition conditions. Intra-class variability is substantial across all categories due to differences in illumination. For example, pollen cells exhibit wide color variation, while capped brood cells vary in texture and shading depending on developmental stage and lighting conditions. Intra-class variability is also reflected in the observed distributions of object size and image brightness, which capture variability in biological structure and imaging conditions.

### 3.4. YOLO Tuning

We tuned the YOLO models on a workstation equipped with an NVIDIA GeForce RTX 2080 Ti GPU running Ubuntu 22.04.5 LTS and Python 3.10.12. The GeForce RTX 2080 Ti is a GPU with 2944 CUDA cores and approximately 10.9 GB of GDDR6 VRAM. During the iterative dataset growth phase (September 2024–December 2025), tuning cycles were performed on 80/20 random splits to monitor model behavior as annotated volume increased. After image annotation was frozen on 31 December 2025, a final 80/20 split was generated and preserved in the files train.txt and valid.txt included in the distribution. All benchmark results reported in Section 4 correspond to this split. All YOLO tuning experiments (YOLOv8n, YOLOv8s, YOLOv11n, and YOLOv11s) were conducted using a shared hyperparameter configuration to enable consistent cross-model benchmarking (cf. Table A1 in Appendix A for the description and rationale of our tuning parameters). E.g., the script

from ultralytics import YOLO
y8n = YOLO('y8n_best.pt')
tune_results = y8n.tune(data='data.yaml', augment=True,
                        hsv_h=0.015, hsv_s=0.7, hsv_v=0.4,
                        degrees=5.0, shear=0.1, translate=0.1,
                        scale=0.5, flipud=0.5, fliplr=0.5,
                        imgsz=1280, patience=10, device=0,
                        batch=5)
shows how we tuned YOLOv8n. We tuned the other three architectures with analogous scripts, all of which are included in the distribution along with the weights of the best models obtained with them. Model tuning was performed iteratively. Beginning in September 2024, newly annotated images were incorporated on a roughly monthly basis, after which all YOLO configurations (YOLOv8n/s and YOLOv11n/s) were re-tuned starting from the best-performing weights of the previous cycle. This incremental protocol continued until 31 December 2025, when image annotation was frozen for manuscript preparation.

We also tuned the medium-capacity YOLOv8m and YOLOv11m architectures. However, once the dataset approached 1000 annotated images, the tuning became unstable and was discontinued. In particular, repeated tuning tests with YOLOv8m and YOLOv11m under the same configuration (10.9 GB of GDDR6 VRAM, imgsz = 1280, batch size = 5) revealed sustained memory limitations. The output below shows the typical first three iterations of tuning YOLOv8m and YOLOv11m.

Epoch  GPU_mem  box_loss  cls_loss  dfl_loss  Instances  Size
1/100  8.96G    3.766     6.046      2.2       905        1280:  0% 1/203  
[WARNING: CUDA OutOfMemoryError in TaskAlignedAssigner, using CPU
1/100  10.4G    3.738     5.587      2.173     1069       1280:  5% 10/203 
[WARNING: CUDA OutOfMemoryError in TaskAlignedAssigner, using CPU
1/100  9.73G    3.729     5.498      2.141     1584       1280:  5% 11/203 
[WARNING: CUDA OutOfMemoryError in TaskAlignedAssigner, using CPU
1/100  9.73G    3.71      5.317      2.11      716        1280:  7% 14/203 


The immediate fallback to CPU execution resulted in degraded and inconsistent tuning behavior rather than stable GPU-based optimization. Across the iterative consensus-based image annotation and model tuning process, well over one hundred major tuning cycles and several hundred tuned model instances were obtained. The four models reported in Section 4 correspond to the best-performing configurations under the shared hyperparameter protocol described in Table A1 in Appendix A.

### 3.5. FAIR Principles

The FAIR data principles provide a framework for ensuring that scientific datasets are accompanied by sufficient contextual metadata to support discovery, interpretation, and reuse. Introduced by Wilkinson et al. [40], and further elaborated in subsequent work [41,42], FAIR emphasizes persistent identifiers, adequate documentation, transparent provenance, open formats, and clear licensing. In precision apiculture, where colony health is shaped by complex environmental, chemical, and management variables, contextualized metadata is essential for enabling reproducible benchmarking and cross-investigation and cross-model comparison. Below we describe our efforts to integrate FAIRness into FAIRHiveFrames-1K.

**Findability:** FAIRHiveFrames-1K is distributed as a self-contained zip archive with a consistent directory structure and clearly separated components for annotation, training, and reproducibility. To promote findability to global audiences interested in precision apiculture, we published the DOI, the version, and the SHA256 checksum of the dataset on zenodo.org under CC-BY 4.0 license [43]. We described the USDA-ARS reservoir and our data curation efforts at USDA Ag Data Commons (data.nal.usda.gov) [39]. Operated by the National Agricultural Library (NAL), Ag Data Commons is the primary discovery portal for all datasets and associated metadata produced by USDA-funded projects. To promote our data engineering efforts to the local beekeeping and horticulture communities, we announced our project at the USU Extension Thriving Hives Program site [44]. USU Extension has developed this certification program in response to high percentages of Utah colonies lost to Varroa.

**Accessibility:** FAIRHiveFrames-1K is registered at Zenodo and hosted on Utah State University’s Box storage infrastructure, which provides continuous, web-based access to the dataset without the need for specialized credentials, proprietary software, or institutional subscriptions. The dataset is downloadable with standard web browsers and command-line tools, enabling straightforward access for researchers, practitioners, citizen scientists, and educators.

**Interoperability:** FAIRHiveFrames-1K uses standard file formats. All images are provided as lossless PNG files, and annotations are provided in YOLO plaintext and YAML to support immediate use with Ultralytics training workflows. The data directory contains two format-conversion scripts: txt_to_xml_converter.py and xml_to_txt_converter.py. The former converts YOLO plaintext to labelImg-compatible, PascalVOC XML to facilitate reuse with alternative annotation tools and PascalVOC XML-compatible models; the latter converts PascalVOC XML to YOLO-compatible plaintext. We also provide the file data.yaml in case potential users want to add their own categories.

**Reusability:** FAIRHiveFrames-1K is distributed with ready-to-run resources for data engineering and model benchmarking. The src directory contains the scripts we used in dataset preparation and model training. This directory includes our utility for splitting training and validation sets (train_valid_split.py) and four model-specific tuning scripts (tune_y8n.py, tune_y8s.py, tune_y11n.py, and tune_y11s.py). The runs directory contains the tuning artifacts of the four best models including their confusion matrices, F1 scores, and best and last weights (best.pt and last.pt). The yolo_intro directory contains the necessary project files that educators can quickly adapt for their courses or tutorials. We added this feature to facilitate the dataset’s reuse as a teaching resource for precision apiculture, scientific computing, and machine learning.

## 4. Results

### 4.1. YOLO Precision, Recall, and mAP

Table 7 summarizes the best validation results obtained using the four YOLO architectures at an input resolution of 1280 × 1280. Lower input resolutions (320 × 320 and 640 × 640) produced weaker results, while 2560 × 2560 resulted in CUDA out-of-memory errors on our hardware. Across all models, validation precision ranged from 0.322 to 0.421, recall from 0.406 to 0.491, mAP@0.50 from 0.334 to 0.392, and mAP@0.50:0.95 from 0.233 to 0.287.

YOLOv11n achieved the highest precision (0.421) and recall (0.491), while attaining mAP values comparable to YOLOv8n (mAP@0.50: 0.390 vs. 0.392; mAP@0.50:0.95: 0.284 vs. 0.287). Thus, the two nano configurations serve as practical conservative baselines for hive-frame cell detection under modest GPU memory constraints. The small-capacity variants (YOLOv8s and YOLOv11s) did not outperform their nano counterparts in terms of mAP@0.50:0.95. Although these models provide increased representational capacity, their performance remained limited under dense cell-level detection conditions. The reason is that under COCO-style evaluation, detections are counted as true positives only if their IoU (Intersection-over-Union) with an explicitly labeled ground-truth instance exceeds a specified threshold (e.g., 0.50 for mAP@0.50 and multiple thresholds from 0.50 to 0.95 for mAP@0.50:0.95). In images containing small numbers of annotated objects and large numbers of unannotated objects, biologically correct detections are considered false positives, because they do not match explicitly labeled instances. Thus, the reported mAP values reflect conservative baselines under sparse labeling.

### 4.2. Per-Category Performance and Confusion Patterns

Table 8, Table 9, Table 10 and Table 11 summarize per-category precision, recall, and F1 scores derived from the confusion matrices of the four YOLO architectures. The full confusion matrix visualizations (raw and normalized) are included in the runs directory of each model in the dataset distribution.

Across all four architectures, *BeeHiveFrame* exhibits consistently strong performance (precision and recall near 1.0). This is expected, as *BeeHiveFrame* corresponds to a large, visually coherent object occupying a substantial image region with exactly one annotated category instance per image, making localization comparatively straightforward. In contrast, all cell-level categories show substantially lower precision and recall. Among the cell-level categories, *UncappedWorkerLarvaCell* achieves the strongest and most stable performance across the tuned models. Its comparatively distinctive visual appearance likely facilitates separability from *CappedWorkerBroodCell* and *EmptyCombCell*. On the other hand, the visually similar category pairs *CappedHoneyCell* vs. *CappedWorkerBroodCell* remain difficult to disentangle under current annotation density and COCO-style evaluation.

*EmptyCombCell* exhibits a particularly instructive detection pattern: recall is moderate to high, while precision is often low (especially for YOLOv8s and YOLOv11s). This behavior is consistent with the practical impossibility of exhaustively annotating empty cells in every image, because a single image may contain hundreds of empty comb cells. Consequently, under standard object detection evaluation, detections of unlabeled but visually valid empty comb cells are counted as false positives, artificially depressing precision, because these detections are counted as *Background*. For dense cell-level categories, therefore, precision should be interpreted as a conservative baseline under incomplete instance-level annotation, and per-category comparisons are most meaningful in relative rather than absolute terms. In sum, all four models reveal a consistent elevation of false-positive counts of sparsely annotated cell-level categories, which is reflected in large counts in *Background*.

### 4.3. Qualitative Analysis

**Figure 4:**
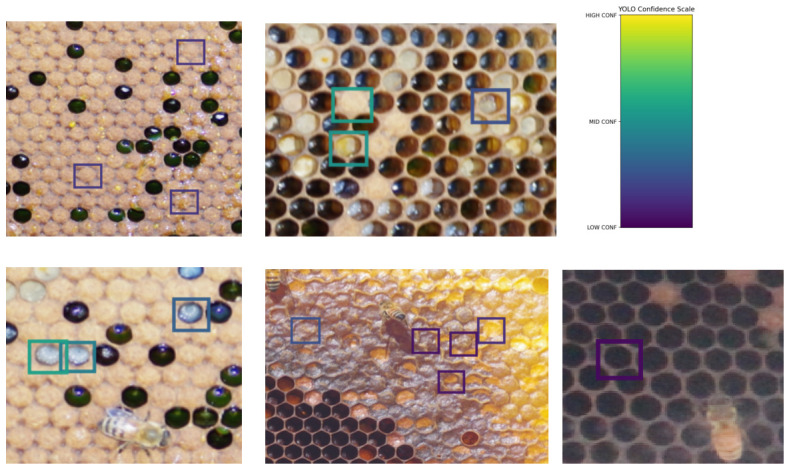
**Representative detection outcomes on FAIRHivesFrames-1K. Top left**: Correct low-confidence detection of multiple *CappedWorkerBroodCell* instances (true positives); **Top middle**: High-confidence detection (top green rectangle on the left) is false positive, i.e., *CappedWorkerBroodCell* is classified as *PollenCell*, the other two detections (one high-confidence, one low-confidence) are correct detections of *PollenCell* (true positives); **Top right**: Confidence color bar to estimate high- and low-confidence detections. **Bottom left**: Correct high- and low-confidence detections of *UncappedWorkerLarvaCell* (true positives); **Bottom middle**: Correct high- and low-confidence detections of *CappendHoneyCell* (true positives); **Bottom right**: Correct low-confidence detection of *EmptyCombCell* (true positive).

To complement the quantitative evaluation with a qualitative analysis, we provide representative qualitative examples of YOLO model predictions on validation images in Figure 4. These examples illustrate typical detection behavior. Correct detections are commonly observed for visually distinctive categories such as *BeeHiveFrame* and *UncappedWorkerLarvaCell*, where structural features are pronounced. Qualitative inspection suggests that, among the labeled categories, recall is high. The dominant source of errors is false positives, in that most labeled instances are successfully detected, while incorrect predictions arise primarily from detections of unlabeled but visually valid cells or from misclassifications among similar categories.

## 5. Discussion

### 5.1. Exhaustive Cell-Level Labeling and Ontological Expansion

**Figure 5:**
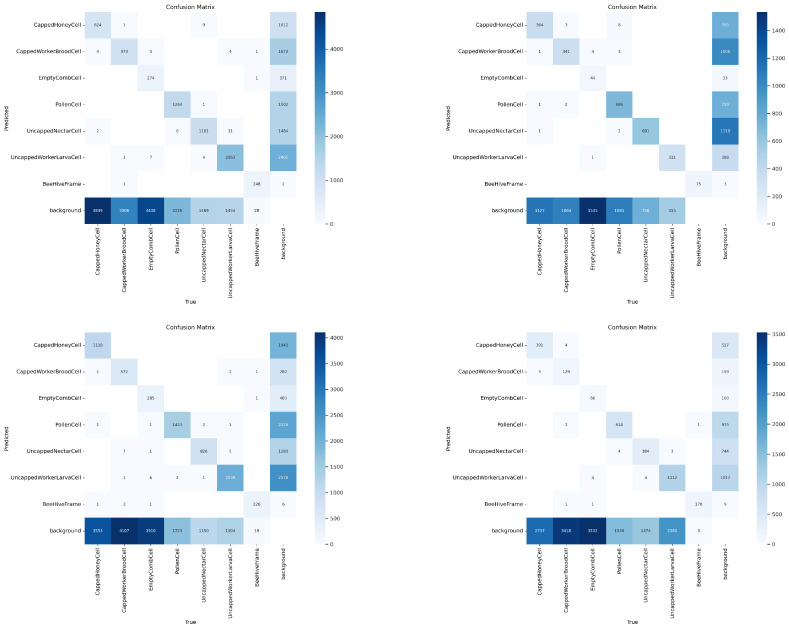
**COCO-style confusion matrices of best YOLO models on the validation set of FAIRHiveFrames-1K.** Row 1: YOLOv8n (left); YOLOv8s (right); Row 2: YOLOv11n (left); YOLOv11s (right). Note the low *Background* counts for *BeeHiveFrame* compared to the cell-level categories. *BeeHiveFrame* is the only category whose singleton instance is annotated in every image.

A limitation of applying standard COCO-style object detection evaluation to cells in hive frame images is the implicit assumption of exhaustive cell-level labeling. In dense hive frame scenes, this assumption is difficult to satisfy: a single 1920 × 1080 frame image may contain hundreds of empty, nectar, pollen, and brood cells. Thus, detections corresponding to biologically valid but unlabeled hive frame cell instances are scored as false positives against *Background* under standard IoU matching rules. This effect artificially suppresses precision and inflates confusion matrix counts attributed to *Background* (cf. Figure 5). As a result, the benchmarks reported in this article should be interpreted as conservative baselines under incomplete cell-level labeling rather than definitive estimates of achievable biological detection quality in automated comb analysis.

Our quantitative analysis confirms that FAIRHiveFrames-1K is dominated by small-object instances, with typical cell-level bounding boxes measuring approximately 25–30 pixels per side. In such settings, even small deviations in predicted bounding boxes can lead to significant penalties under IoU-based evaluation metrics. Furthermore, since we had no control over the acquisition of the original images in the field, standard data-centric strategies such as diversity-driven sampling or core-set selection are not straightforward to apply. These approaches typically assume control over original data acquisition. That said, while the dataset construction process was not explicitly guided by formal data-centric methodologies, the iterative incorporation of newly annotated images over time, combined with repeated base model re-tuning, provided an implicit form of progressive, consensus-based dataset refinement.

Our longitudinal, iterative curation of FAIRHiveFrames-1K has convinced us that there is a need for additional biologically meaningful annotation categories such as *QueenCell*, *QueenCup*, *CappedDroneBroodCell*, and *Bee*. The first three categories have clear relevance to colony health assessment in standard hive inspections [6]. Our hypothesis is that explicitly modeling the fourth category *Bee* will improve cell-detection robustness by accounting for bee-occupied regions that occlude individual cells. In other words, treating bees as first-class objects will reduce spurious detections in dense hive frame scenes. Our future work will, therefore, focus on expanding our annotation category repertoire with these four categories.

### 5.2. Empirical Confidence Calibration for Dense Hive Frame Detection

A range of approaches has been proposed in the literature to address small-object detection challenges [45,46], including multi-scale training strategies, feature pyramid enhancements, and slicing-based inference methods such as Slicing Aided Hyper Inference (SAHI) [47], which partitions high-resolution images into smaller regions to improve detection sensitivity. While these approaches are promising, our work focuses on establishing a FAIR dataset for image-based comb analysis decoupled from specific object classification models. The YOLO models are provided only as preliminary conservative baseline benchmarks. The used annotation formats make the dataset accessible to other models as well as to the exploration and exploitation of specialized cell detection and cell detection calibration techniques. These are important directions of our future research.

Specifically, in dense hive frame images, object detection models such as YOLO produce bounding boxes with associated confidence scores. However, our analysis indicates that these confidence scores are not well calibrated and do not reliably reflect the probability of correctness, particularly in low-confidence regions and for visually ambiguous categories. As a result, COCO-style evaluation systematically penalizes detections of unlabeled but visually valid cells as false positives, leading to suppressed precision despite high recall of labeled instances.

To address this limitation without altering the underlying COCO-style evaluation protocol, we are formulating a probability-theoretic confidence calibration method that treats model outputs as measurements with uncertainty. The approach estimates class- and confidence-conditioned empirical precision via stratified sampling and subsequent human validation. It is important to note here that human validation in this approach does not imply any changes to our consensus-based annotation method.

Let c∈C denote a class (e.g., *CappedHoneyCell*) and b∈B a confidence bin. For each pair (c,b), we define empirical precision as:

$${\hat{p}}_{c,b}=\frac{N_{c,b}^{\mathrm{correct}}}{N_{c,b}^{\mathrm{correct}}+N_{c,b}^{\mathrm{incorrect}}}$$

where $N_{c,b}^{\mathrm{correct}}$ and $N_{c,b}^{\mathrm{incorrect}}$ are obtained from human evaluation of stratified samples of existing detections. Given a detection *i* with class *c*, confidence bin $b_{i}$, and predicted area $A_{i}$, a calibrated estimate is computed as:

$$A_{i}^{\mathrm{cal}}={\hat{p}}_{c,b_{i}}\cdot A_{i}$$

and aggregated over existing detections to obtain calibrated measurements:

$$A^{\mathrm{cal}}=\sum_{i}{\hat{p}}_{c,b_{i}}\cdot A_{i}.$$

In this probability-theoretic formulation, empirical precision acts as a probabilistic weight that downscales unreliable detections while preserving high-confidence predictions. What is fundamental is that this approach does not modify COCO-style evaluation, which remains a standardized and reproducible baseline. Instead, our method introduces a calibration layer that improves the interpretability and reliability of existing model outputs in dense and partially annotated settings.

Unlike exhaustive instance-level annotation, which is difficult to scale for images containing hundreds of small and visually similar cells, empirical calibration provides a cost-effective and statistically grounded alternative. It separates geometric detection from uncertainty estimation and allows aggregate measurements to be corrected without requiring complete annotation. We view this calibration layer as a promising extension for deploying object detection models in dense biological domains, where exhaustive annotation is impractical at scale.

### 5.3. Empirical Calibration vs. Pseudo-Labeling

Pseudo-labeling is a semi-supervised learning technique in which model predictions on unlabeled data are treated as ground truth and incorporated into subsequent training cycles. In its canonical form [48], pseudo-labeling assigns to each input *I* the label

$$\hat{L}=\arg\max_{L}p\left(L|I\right)$$

and retains predictions above a fixed confidence threshold. These pseudo-labels are introduced to the dataset as true labels in an iterative self-training loop [49].

Pseudo-labeling relies on two critical assumptions of confidence reliability and high-confidence filtering. The former states that model confidence scores are well-calibrated approximations for correctness. The latter states that low-confidence predictions are unreliable and should be discarded. Our empirical analysis of YOLO performance on FAIRHiveFrames-1K indicates that both assumptions do not hold for dense hive frame images for the following reasons.

First, confidence is not a reliable predictor of correctness, because low-confidence detections frequently exhibit high empirical precision for several categories. This observation appears to be consistent with recent findings that confidence-based selection fails when correct and incorrect predictions overlap in score distributions [50].

Second, confidence-based filtering leads to systematic loss of valid classification. In dense hive frame images, a single frame may contain hundreds of visually valid cells, many of which are not exhaustively annotated due to the prohibitive cost of manual labeling. Consequently, low-confidence predictions often correspond to biologically valid but unlabeled instances. Discarding these predictions will likely remove a substantial portion of correct detections and introduce selection bias [50].

Third, pseudo-labeling introduces a feedback loop that amplifies model bias. When treated as ground truth, incorrect pseudo-labels are reinforced during retraining, which leads to confirmation bias and error propagation [51]. This effect is especially problematic in dense hive frame images, where subtle visual differences between categories increase the likelihood of systemic misclassification.

Finally, pseudo-labeling implicitly assumes that model confidence is calibrated. However, modern deep neural networks are known to produce poorly calibrated confidence estimates [52], which further undermines the validity of confidence-thresholded pseudo-labels.

Our empirical calibration method briefly outlined in the previous section does not treat model predictions as ground truth. Prediction uncertainty is explicitly modeled by estimating category- and confidence-conditioned empirical precision. Each detection is weighted probabilistically rather than converted into a binary label. This formulation does not assume confidence calibration or require exhaustive labeling. All detections contribute proportionally, and low-confidence predictions are never discarded. Most importantly, our formulation does not introduce a self-training loop, because detection and calibration are completely decoupled. A potential limitation of our method is the requirement of periodic recalibration that involves at least two human annotators of stratified samples of existing model detections.

## 6. Conclusions

Only time will determine the extent to which FAIRHiveFrames-1K achieves FAIRness. Our design decisions were intended to lower entry barriers for researchers, practitioners, citizen scientists, and educators seeking to automate image-based hive frame analysis. In our investigation, we adopted a transparent baseline strategy (we will call it Option A): iterative, consensus-based image annotation coupled with iterative base model re-tuning under standard COCO-style evaluation. We gradually discovered that under sparse cell-level labeling, many cell-level detections scored as false positives correspond to biologically valid but unlabeled cells, especially in dense categories such as *EmptyCombCell* and *CappedWorkerBroodCell*. Therefore, our reported model tuning results provide conservative lower-bound cell-detection baselines. A more labor-intensive alternative (Option B) is the construction of evaluation subsets consisting of fully annotated hive frame image regions or entire hive frame images. Exhaustive labeling of such regions or images, if humanly feasible, may enable more accurate precision and recall estimates under standard COCO-style evaluation, though at substantially increased annotation cost. For cell categories where instance-level annotation is impractical at scale (e.g., hundreds of empty comb or uncapped nectar cells per image), alternative task formulations may be more appropriate (Option C). These include region-level or aggregate labeling strategies such as semantic segmentation or, which is what our focus is, and will be in the near future, empirical detection confidence calibration. Such approaches may better align evaluation with the biological structure of the hive frame.

From a strict computer vision perspective, FAIRHiveFrames-1K presents a dense small-object detection challenge, where hundreds of visually similar small instances co-occur within a single image. Beyond object detection, FAIRHiveFrames-1K can be viewed as a component of a broader Agriculture 4.0 ecosystem for managed honey bee colonies, because digital imaging will increasingly complement standard hive inspections or standard colony monitoring measurements (e.g., hive weight, in-hive temperature, and in-hive CO_2_) on edge or cloud-based AI systems.

## Figures and Tables

**Table 1:** **High-level comparison between DeepBee© [18] and FAIRHiveFrames-1K.**

Feature	DeepBee (2020)	FAIRHiveFrames-1K
Public image dataset	Classification/segmentation datasets publicly available	Annotated PNG images publicly available under CC-BY 4.0 license
Annotation format	Custom: (classification: id, x, y, radius; segmentation: mask images)	Standard: PascalVOC XML; YOLO plaintext
Pretrained weights	Publicly available (Google Drive/GitHub)	Publicly available under CC-BY 4.0 (Zenodo)
Dataset splits	Train/test CSVs; retraining involves random splitting	Fixed 80/20 split included in distribution
Versioning	Not explicitly versioned	Included with dataset release
FAIR alignment	Not explicitly framed in FAIR terms	Explicitly designed under FAIR principles
Illumination, background, distance conrol	Strong	Minimal/None
Model/data decoupling	Minimal	Complete
Primary emphasis	Integrated deep learning pipeline for comb analysis	Dataset engineering, reproducible baselines for comb analysis

**Table 2:** **CHBRC publications on field experiments (2013–2022) that generated the USDA–ARS reservoir underlying FAIRHiveFrames-1K.** Location abbreviations: AZ—Arizona; MS—Mississippi; AS—Arkansas; ND—North Dakota; ID—Idaho; CA—California; CRP—Conservation Reserve Program.

Reference	Location	Research Subject	Period
[22]	AZ	Bee Mass, Brood Production	2013–2014
[23]	AZ, MS, AS	Imidacloprid Impact	2014, 2015
[24]	ND, ID, CA	Overwintering	2014–2016
[25]	AZ	Imidacloprid Exposure Quantification	2013–2015
[26]	AZ, CA	Supplemental Forage and Microbiome	2015–2016
[27]	AZ, CA	Pre-Almond Supplemental Forage	2015–2016
[28]	AZ	Breakfast Canyon	2015–2016
[29]	AZ, CA, Australia	Environmental Impact	2013–2017
[30]	ID, CA	Proximity to US CRP lands	2014–2015
[31]	AZ	Methoxyfenozide Impact	2016–2018
[32]	AZ, Australia	Exposure to Chemical Stress	2016
[33]	CA	Landscape Factors	2016–2018
[34]	AZ	Clothianidin Impact	2016–2018
[35]	AZ	Exposure to Flonicamid	2019, 2021
[36]	AZ	Exposure to Neonicotinoid	2014–2019
[37]	AZ	Hive Entrance Orientation	2018–2020
[38]	AZ	Cold Storage	2020–2022

**Table 3:** **Visual characteristics of seven annotated categories in FAIRHiveFrames-1K.**

Category	Visual Characteristics
CappedHoneyCell	Cells sealed with light- or dark-colored wax caps, typically convex and uniform in appearance.
CappedWorkerBroodCell	Cells containing developing worker pupae, sometimes covered with more matte caps compared to honey cells.
EmptyCombCell	Open cells with no visible contents but with noticeable color variation.
PollenCell	Cells with pollen, often with granular texture and color variation (yellow, orange, or mixed hues).
UncappedNectarCell	Open cells with liquid nectar, often with reflective, glossy surface.
UncappedWorkerLarvaCell	Cells, typically white, with visible larvae.
BeeHiveFrame	Full wooden frame structure containing comb cells.

**Table 4:** **Location abbreviations in the names of the images in FAIRHiveFrames-1K.** Table legend: AZ—Arizona; ID—Idaho.

Abbreviation	Location
CHBRC	Carl Hayden Bee Research Center, Tucson, AZ, USA
MAC	University of Arizona Maricopa Agricultural Center, Maricopa, AZ, USA
SRER	Santa Rita Experimental Range, Tucson, AZ, USA
RR	Red Rock Agriculture Center, Red Rock, AZ, USA
HOOPS	CHBRC apiary in Tucson, AZ, USA
SC	Shipping Corral, AZ, USA
BC	Bear Cage, an internal CHBRC apiary site in Tucson, AZ, USA
CT	Cow Town, ID, USA

**Table 5:** **Counts of the annotated regions of interest (ROIs) by category in FAIRHiveFrames-1K.**

Category ID	Category Label	Num. Annotated ROI
0	CappedHoneyCell	25,422
1	CappedWorkerBroodCell	24,966
2	EmptyCombCell	23,677
3	PollenCell	18,078
4	UncappedNectarCell	12,119
5	UncappedWorkerLarvaCell	19,142
6	BeeHiveFrame	1265
**TOTAL**		**124,669**

**Table 6:** **Bounding box areas in FAIRHiveFrames-1K.** All values are in pixels^2^.

Category	Mean	Median	Std
All Objects	12,971	625	124,486
Cell-Level (Classes 0–5)	633	624	159
BeeHiveFrame (Class 6)	1,216,507	1,267,110	252,753

**Table 7:** **Preliminary benchmark results for YOLOv8 and YOLOv11 architectures tuned on FAIRHiveFrames-1K at an input resolution of 1280 × 1280.**

Architecture	Precision	Recall	mAP@0.50	mAP@0.50:0.95
YOLOv8n	0.389	0.473	0.392	0.287
YOLOv8s	0.398	0.406	0.349	0.243
YOLOv11n	0.421	0.491	0.390	0.284
YOLOv11s	0.322	0.441	0.334	0.233

**Table 8:** **Per-category performance summary for YOLOv8n on FAIRHiveFrames-1K (imgsz = 1280).**

Category	Precision	Recall	F1
CappedHoneyCell	0.182	0.437	0.257
CappedWorkerBroodCell	0.292	0.336	0.312
EmptyCombCell	0.077	0.392	0.129
PollenCell	0.401	0.447	0.423
UncappedNectarCell	0.350	0.429	0.386
UncappedWorkerLarvaCell	0.573	0.422	0.486
BeeHiveFrame	0.954	0.987	0.970

**Table 9:** **Per-category performance summary for YOLOv8s on FAIRHiveFrames-1K (imgsz = 1280).**

Category	Precision	Recall	F1
CappedHoneyCell	0.244	0.319	0.276
CappedWorkerBroodCell	0.242	0.252	0.247
EmptyCombCell	0.028	0.571	0.053
PollenCell	0.389	0.477	0.429
UncappedNectarCell	0.443	0.349	0.390
UncappedWorkerLarvaCell	0.359	0.444	0.397
BeeHiveFrame	1.000	0.962	0.980

**Table 10:** **Per-category performance summary for YOLOv11n on FAIRHiveFrames-1K (imgsz = 1280).**

Category	Precision	Recall	F1
CappedHoneyCell	0.239	0.365	0.289
CappedWorkerBroodCell	0.122	0.422	0.189
EmptyCombCell	0.068	0.382	0.115
PollenCell	0.452	0.390	0.419
UncappedNectarCell	0.428	0.399	0.413
UncappedWorkerLarvaCell	0.608	0.441	0.511
BeeHiveFrame	0.915	0.958	0.936

**Table 11:** **Per-category performance summary for YOLOv11s on FAIRHiveFrames-1K (imgsz = 1280).**

Category	Precision	Recall	F1
CappedHoneyCell	0.125	0.429	0.193
CappedWorkerBroodCell	0.036	0.440	0.067
EmptyCombCell	0.018	0.398	0.035
PollenCell	0.287	0.390	0.331
UncappedNectarCell	0.218	0.338	0.265
UncappedWorkerLarvaCell	0.337	0.520	0.409
BeeHiveFrame	0.977	0.939	0.958

## Data Availability

FAIRHiveFrames-1K Version 1.0 is publicly available under a CC-BY 4.0 license at https://doi.org/10.5281/zenodo.19241078. All images included in FAIRHiveFrames-1K are provided in their entirety as PNG files in the data/obj/ directory, along with corresponding annotation files, making the dataset fully self-contained. The SHA256 checksum of the dataset zip archive is 280ad37fda41a90d19b79b9339da49504685d83c3f57a7c2f7b8d44578e0ef15. The dataset can also be downloaded from Utah State University Box at: https://usu.box.com/shared/static/kzmjdnzesdf4ssg5zpo5w6xiyycjz7y3.zip (accessed on 11 April 2026).

## Appendix A

**Table A1:** **Shared hyperparameters used in tuning YOLO models on FAIRHiveFrames-1K.**

Parameter	Value	Description and Rationale
augment	True	Enables training-time data augmentation to improve generalization across variations in lighting, viewpoint, comb appearance.
hsv_h	0.015	Hue jitter applied in HSV color space; small value preserves biological color realism while improving robustness to color variation.
hsv_s	0.7	Saturation jitter; supports robustness to varying illumination and exposure conditions in hive frame imagery.
hsv_v	0.4	Brightness (value) jitter; accounts for shadows, glare, and lighting heterogeneity across apiaries.
degrees	5.0	Random rotation (±5°) simulating realistic camera tilt during hive inspections without excessive geometric distortion.
shear	0.1	Shearing transformation to model perspective skew introduced by handheld photography.
translate	0.1	Random image translation (up to 10%) promoting spatial invariance of object detection across the frame.
scale	0.5	Random scaling to simulate variability in camera distance and effective cell size.
flipud	0.5	Random vertical flipping; used to encourage orientation invariance in cell-level detection.
fliplr	0.5	Random horizontal flipping; reflects symmetry in hive frame photography and handling.
imgsz	1280	Input image resolution; selected to preserve fine-grained comb-cell structure while remaining feasible under GPU memory constraints.
batch	5	Training batch size; selected to accommodate high-resolution input images on an RTX 2080 Ti GPU with 11 GB VRAM.
patience	10	Early stopping patience; terminates training if validation performance does not improve for 10 evaluation cycles.
device	0	Specifies use of the first available GPU device for training and evaluation.
